# Survival outcomes of the patients with advanced laryngeal squamous cell carcinoma treated with chemoradiotherapy and total laryngectomy based on reports of head and neck cancer registry of Japan

**DOI:** 10.1007/s10147-025-02938-4

**Published:** 2026-05-07

**Authors:** Yuta Yamamura, Yuto Horichi, Tatsuya Furukawa, Hirotaka Shinomiya, Megumi Kitayama, Daisuke Kawakita, Takeshi Kodaira, Munenaga Nakamizo, Seiichi Yoshimoto, Ken-ichi Nibu

**Affiliations:** 1 Department of Otolaryngology-Head and Neck Surgery, Kobe University School of Medicine, 7-5-1 Kusunoki-cho, Chuo-ku, Kobe, 650-0017 Japan; 2 Clinical Study Support Center, Data Center Department, Wakayama Medical University Hospital, Wakayama, Japan; 3 Department of Otorhinolaryngology-Head and Neck Surgery, Nagoya City University Graduate School of Medical Sciences, Nagoya, Japan; 4 Department of Radiation Oncology, Aichi Cancer Center, Nagoya, Japan; 5 Department of Otorhinolaryngology-Head and Neck Surgery, Tokyo Women’s Medical University, Tokyo, Japan; 6 Department of Head and Neck Surgery, National Cancer Center Hospital, Tokyo, Japan; 7 Japan Society for Head and Neck Cancer, Tokyo, Japan

**Keywords:** Laryngeal squamous cell carcinoma, Head and neck cancer, Chemoradiotherapy, Surgery, Induction chemotherapy

## Abstract

**Background:**

Roles of induction chemotherapy (ICT) and chemoradiotherapy (CRT) for laryngeal preservation in patients with locally advanced laryngeal cancer (LC) remain unclear.

**Patients and methods:**

We enrolled 834 patients with T3N0-2M0 and T4aN0-2M0 LC registered in the Head and Neck Cancer Registry of Japan between 2011 and 2015. Oncological outcomes of total laryngectomy (TL), CRT, and ICT were evaluated. Propensity score-matching analyses (PSMA) were performed between TL and CRT, excluding ICT.

**Results:**

No significant differences were observed in overall survival (OS) and disease-specific survival (DSS) among patients treated by TL, CRT, and ICT followed by TL or CRT, and among patients treated by TL and CRT in PSMA. Of note, no significant difference was observed in LCR and LRFS between TL and ICT groups.

**Conclusions:**

ICT and CRT did not yield survival benefits but might contribute to laryngeal preservation without compromising survival in patients with T3-4aN0-2M0.

**Supplementary Information:**

The online version contains supplementary material available at 10.1007/s10147-025-02938-4.

## Introduction

Laryngeal cancer (LC) is one of the most common head and neck cancers. The prevalence of LC has been decreasing in developed countries owing to a decline in smoking habits. However, it remains common, with > 170,000 new cases being diagnosed annually [1–5]. The main treatment modality for advanced LC is mainly total laryngectomy (TL). However, there has been a recent increase in treatment with chemoradiotherapy (CRT), which allows laryngeal preservation. Although several studies have compared the outcomes of TL and CRT, the optimal treatment strategy for locally advanced LC remains unclear [6–12]. Since it is difficult to perform randomized controlled trials comparing these treatment modalities since the patient’s desire to preserve the larynx crucially contributes to the treatment choice, in this study, we investigated and compared the oncological outcomes of patients with locally advanced LC which were treated with CRT or TL in Japan using data from the Head and Neck Cancer Registry of Japan [13].

## Patients and methods

### Study design

Clinical data of patients newly diagnosed with T3N0-2M0 or T4aN0-2M0 squamous cell carcinoma (SCC) of the larynx between 2011 and 2015 were obtained from the Head and Neck Cancer Registry of Japan. We used the following Histology and Internal Classification of Diseases for Oncology (3rd edition) codes for LC and SCC: C32.9, M8070/2, 8070/3, 8071/3, 8072/3, 8073/3, 8074/3, and 8076/2. We only included patients who underwent TL, total pharyngolaryngectomy, or TL with partial pharyngectomy and those who received CRT as radical therapy. Cancer stage classification was performed using the 7th TNM classification system.

We excluded patients who underwent palliative treatment, radiotherapy (RT) alone, or larynx-sparing surgery. Additionally, we excluded patients with unclear T classifications; patients with T4b and/or N3 classifications; and patients with missing data regarding prognosis, performance status (PS), and/or treatment methods. The primary endpoints were local recurrence-free survival (LRFS), local control rate (LCR), disease-specific survival (DSS), and overall survival (OS). This study was approved by the Institutional Ethical Committee of Kobe University Hospital (# B240006). Informed consents were obtained through an opt-out option on the website.

### Statistical analysis

Patients were classified to patients treated by TL (TL group), patients treated with CRT (CRT group), and patients who received induction chemotherapy (ICT) (ICT group). Patients in ICT group were further classified to patients treated with ICT followed by TL (ICT-TL group) and patients treated with ICT followed by CRT (ICT- CRT group). Chi-square and Student’s t-tests were used to assess potential correlations among patients in each group. Intergroup comparisons were performed for OS, DSS, LRFS, and LCR using the Kaplan–Meier method and log-rank test.

Additionally, a 1:1 ratio propensity score-matched analysis (PSMA) was performed between the TL and CRT groups for both patients with T3N0-2M0 and T4aN0-2M0 using a caliper width of 0.2 logit of standard deviation to allow between-group matching of clinical confounding variables. The propensity scores were calculated using multivariate logistic regression models based on age, sex, and PS. All statistical analyses were performed using R software (Easy R, ver. 4.3.1, The R Project for Statistical Computing).

OS was defined as the duration from the date of treatment initiation to the date of death. DSS was defined as the duration from the date of treatment initiation to the date of death due to LC. In the present study, the only information regarding recurrence was the site of initial recurrence and date of recurrence, with no information regarding subsequent metastases and recurrence sites. Therefore, LRFS was defined as the period from the date of treatment initiation to the date of initial local recurrence or death. The LCR was defined as the period from the date of treatment initiation to the first local recurrence. Patients who underwent surgery immediately after definitive CRT as initial therapy were considered to have undergone salvage surgery for residual tumor after CRT, and the date of completion of CRT was defined as the date of recurrence (the date residual disease was confirmed), with LRFS and LCR defined as one month. Accordingly, their LRFS and LCR were defined as one month. Statistical analyses were performed separately for patients with T3N0-2M0 and T4aN0-2M0.

## Results

**Fig. 1:**
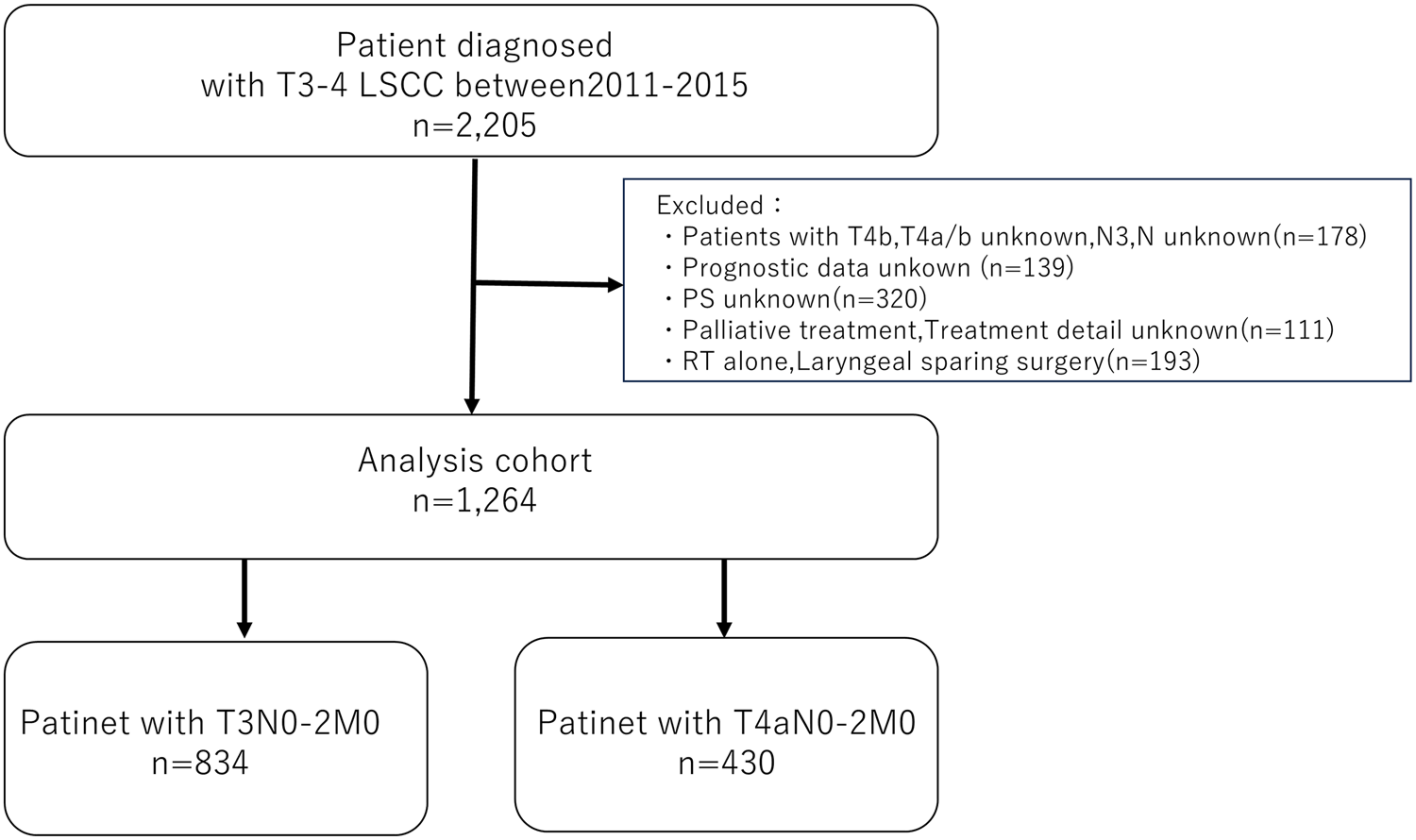
Patient selection flowchart. The Head and Neck Cancer Registry of Japan does not include information regarding the types and doses of chemotherapeutic agents used for CRT. However, based on the Japanese Clinical Practice Guidelines for Head and Neck Cancer, most institutions administer CDDP-based concurrent CRT for advanced laryngeal cancer

Figure 1 shows a flowchart of participant enrollment. Among 2,205 identified patients. We excluded 1,264 patients based on the inclusion/exclusion criteria.

### Patients with T3N0-2M0 laryngeal cancer

There were 834 patients with T3N0-2M0 LC. There were 774 male and 60 females. Median age was 69 years old ranging from 32 to 95 years old. Among them, there were 409, 341, and 84 patients in the TL, CRT, and ICT groups, respectively. There were significant among-group differences in age (*p* < 0.001), sex (*p* = 0.034), PS (*p* < 0.001), clinical N classification (*p* < 0.001) and subsite (*p* < 0.001) (Table 1). Older patients tended to be treated by TL, patients in the CRT group tended to have better PS, and patients in the ICT group tended to have multiple lymph node metastases and supraglottic cancer.

**Table 1:** Characteristics of patients with T3N0-2M0 LC treated with TL, CRT, and ICT before 1:1 propensity score matching

Characteristics	TL (n = 409)	CRT (n = 341)	ICT (n = 84)	*p* value
	No. (%)	No. (%)	No. (%)	
Median age [range] years old	72 [40–95]	67 [32–89]	66 [49–82]	< 0.001
Sex				0.034
Male	370 (90.5)	325 (95.3)	79 (94.0)	
Female	39 (9.5)	16 (4.7)	5 (6.0)	
Performance Status				0.001
0	292 (71.4)	287 (84.2)	67 (79.8)	
1	94 (23.0)	48 (14.1)	16 (19.0)	
2	17 (4.2)	5 (1.5)	0 (0.0)	
3	6 (1.5)	1 (0.3)	1 (1.2)	
cN				< 0.001
N0	286 (69.9)	247 (72.4)	38 (45.2)	
N1	45 (11.0)	31 (9.1)	10 (11.9)	
N2 [a/b/c]	78 (19.1)	63 (18.5)	36 (42.9)	
Subsite				< 0.001
NOS	9 (2.2)	5 (1.5)	0 (0)	
Supraglottic	177 (43.3)	141 (41.3)	57 (67.9)	
Glottic	205 (50.1)	190 (55.7)	26 (31.0)	
Subglottic	18 (4.4)	5 (1.5)	1 (1.2)	

*RT*, radiotherapy; *CRT*, chemoradiotherapy; *TL*, total laryngectomy; *CRT*, chemoradiotherapy; *ICT*, induction chemotherapy, *NOS*, not otherwise specified

**Fig. 2:**
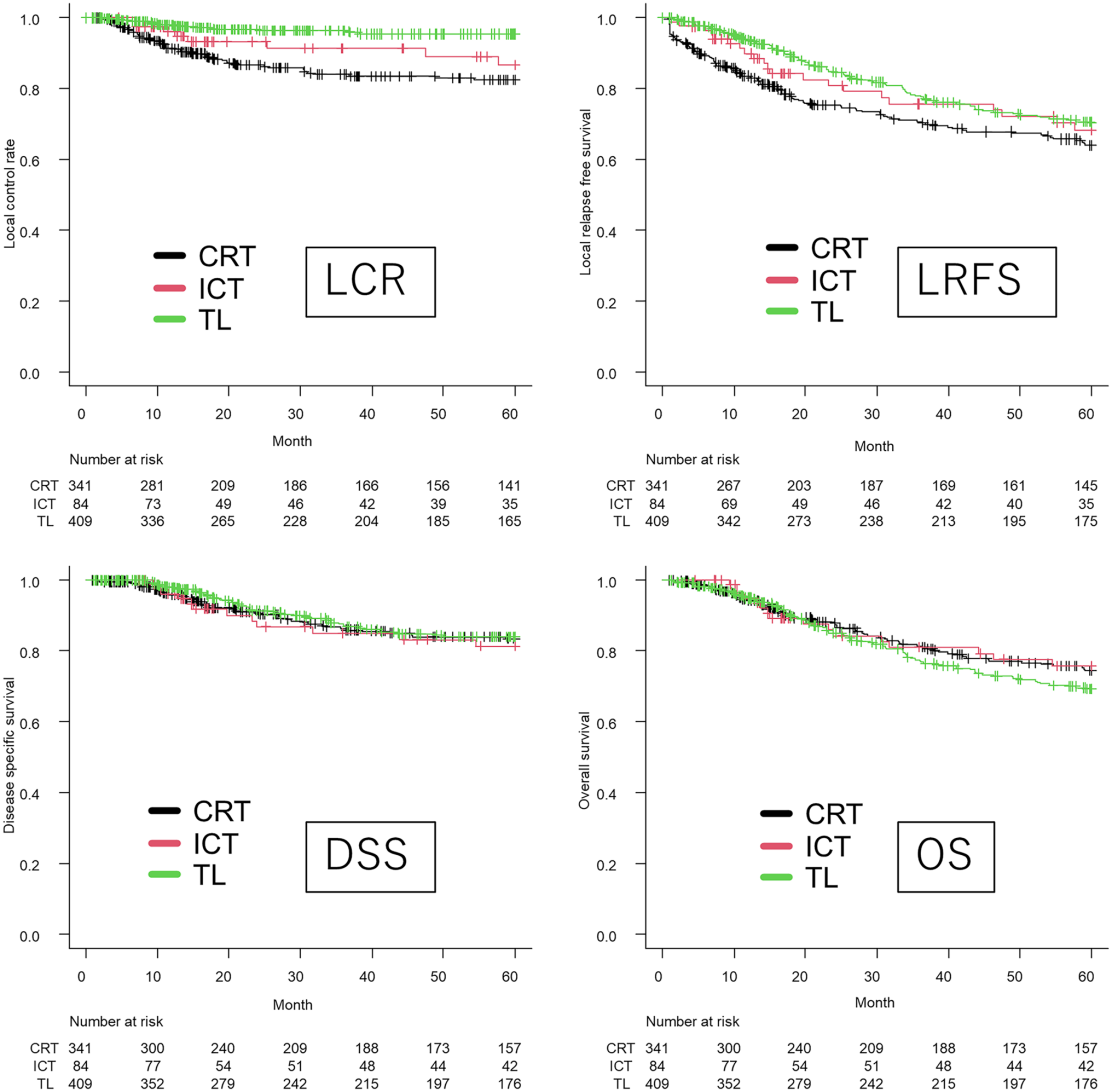
Kaplan–Meier curves of patients with T3N0-2M0 LC treated with TL, CRT, or ICT before 1:1 propensity score matching. CRT, chemoradiotherapy; ICT, induction chemotherapy; LC, laryngeal cancer; TL, total laryngectomy; LCR, local control rate; LRFS, local recurrence-free survival; DSS, disease-specific survival; OS, overall survival. Black line, CRT group; red line, ICT group; light blue line, TL group

Figures 2 shows the outcomes of TL group, CRT group and ICT group in the patients with T3N0-2M0 LC. As shown in Fig. 2, there were no significant differences among the TL, CRT, and ICT groups in the 5-year OS rates (69.3%, 74.5%, 75.9%; *p* = 0.162) and 5-year DSS rates (84.1%, 83.6%, 81.5%; *p* = 0.771). Compared with the CRT group, the TL group had significant better 5-year LRFS rate (70.3% vs. 64.0%, *p* = 0.0493) and 5-year LCR (95.5% vs. 82.4%, *p* < 0.001). While the TL group had a significantly better LCR rate than the ICT group (95.5% vs. 86.6%, *p* = 0.0134), there was no significant difference between the TL and ICT group in the 5-year LRFS rate (70.3% vs. 68.3%, *p* = 0.745). There were no significant differences between the CRT and ICT groups in LRFS (64.0% vs 68.3% *p* = 0.153) and LCR (82.4%vs86.6% *p* = 0.319).

**Fig. 3:**
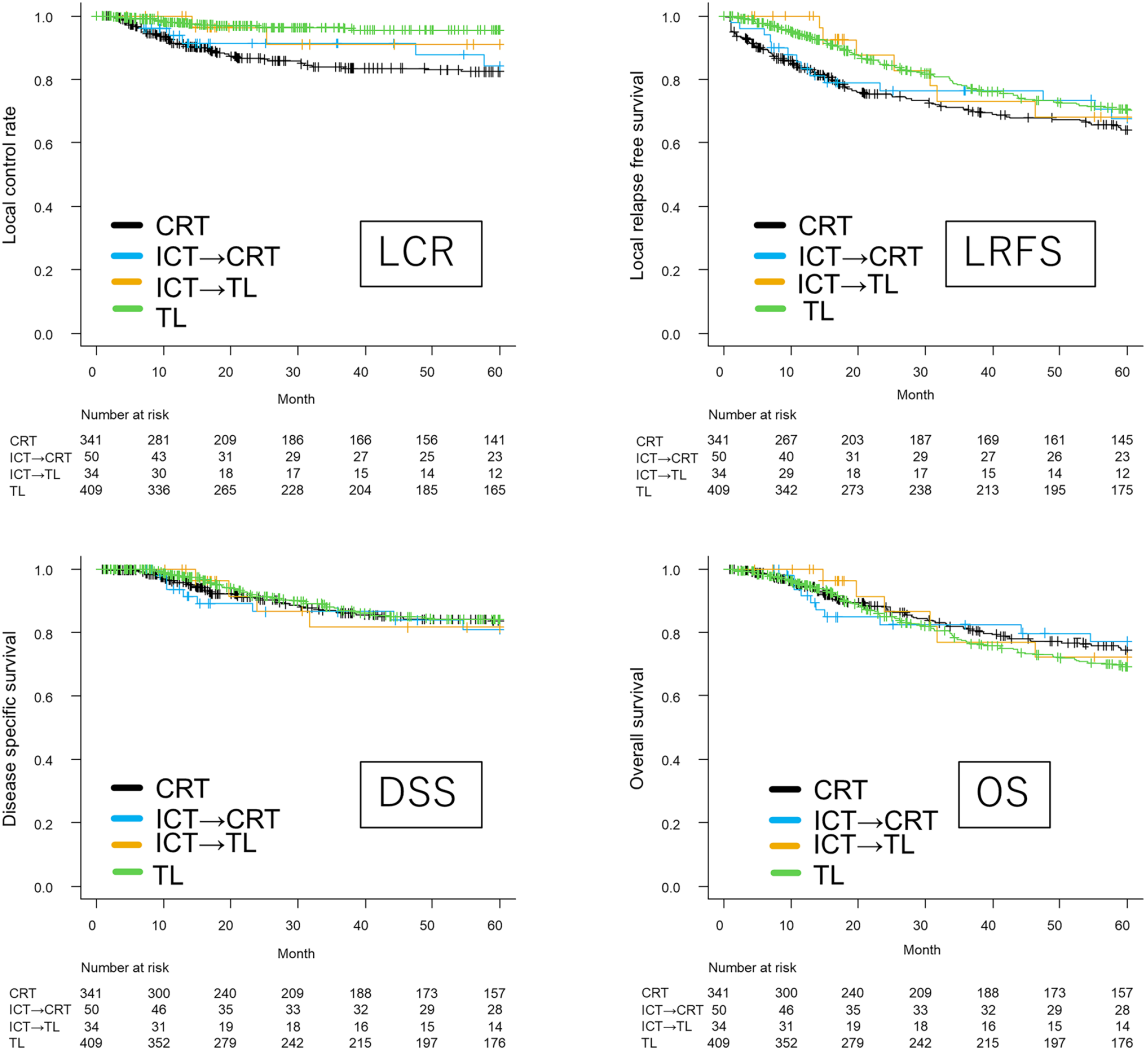
Kaplan–Meier curves of the patients with T3N0-2M0 LC treated with TL, CRT, ICT-TL, or ICT-CRT before 1:1 propensity score matching. CRT, chemoradiotherapy; ICT, induction chemotherapy; LC, laryngeal cancer; TL, total laryngectomy; LCR, local control rate; LRFS, local recurrence-free survival; DSS, disease-specific survival; OS, overall survival. Black line, CRT group; red line, ICT-CRT group; light blue line, ICT-TL group; blue line, TL group

Figure 3 shows the oncological outcomes of the TL, CRT, ICT-TL, ICT-CRT groups in the patients with T3N0-2M0 LC. As shown in Fig. 3, between the TL and ICT-TL groups, there were no significant differences in the 5-year OS rate (69.3% vs. 72.2%; *p* = 0.322), DSS rate (84.1% vs. 91.3%, *p* = 0.907), LCR (95.5% vs. 90.9%, *p* = 0.522), or LRFS (70.3% vs. 68.2%; *p* = 0.448). Similarly, there were no significant differences between the CRT and ICT-CRT groups in the 5-year OS rate (74.5% vs. 77.1%, *p* = 0.998), DSS rate (83.6% vs. 80.9%, *p* = 0.496), LCR (82.4% vs. 84.1%, *p* = 0.802), and LRFS (64.0% vs. 67.6%, *p* = 0.462). Of note, LCR of the ICT-CRT group and ICT-TL group were quite similar.

Because there was a tendency for multiple lymph node metastases to occur in the ICT group, we compared OS and DSS among the TL, CRT, and ICT groups in T3N2M0 cases. There were no significant differences in 5-year OS rate (64.7%, 54.9%, 60.2%; CRT vs ICT *p* = 0.292, TL vs ICT *p* = 0.696) and 5-year DSS rate (63.7%, 63.3%, 68.0%, CRT vs ICT *p* = 0.405, TL vs ICT *p* = 0.88) (supplementary Fig. 1).

**Fig. 4:**
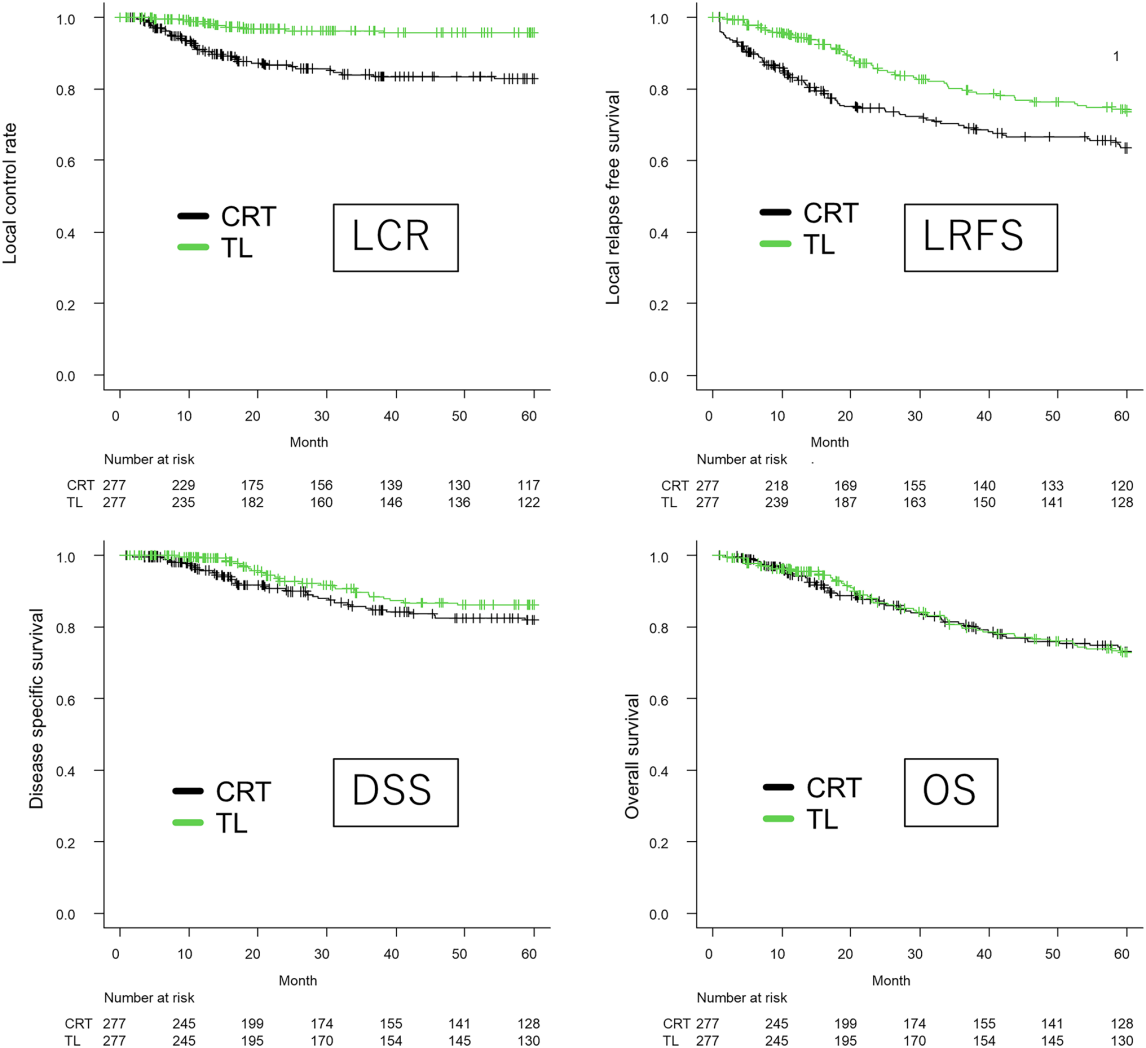
Kaplan–Meier curves of patients with T3N0-2M0 LC treated with TL or CRT after 1:1 propensity score matching. CRT, chemoradiotherapy; TL, total laryngectomy; LCR, local control rate; LRFS, local recurrence-free survival; DSS, disease-specific survival; OS, overall survival. Black line, CRT group; red line, TL group

After excluding the ICT group, we selected 277 pairs using a 1:1 PSMA (Table 2). Figure 4 shows the oncological outcomes of matched patients with T3N0-2M0 LC who underwent TL or CRT. Between the TL and CRT groups, there were significant differences in the 5-year LCR (95.7% vs. 82.8%, *p* < 0.001) and 5-year LRFS (73.7% vs. 63.4%, *p* = 0.00944). However, there were no significant differences in the 5-year OS rate (72.8% vs. 73.1%, *p* = 0.64) and 5-year DSS rate (86.2% vs. 81.9%, *p* = 0.198).

**Table 2:** Characteristics of patients with T3N0-2M0 LC treated with TL and CRT after 1:1 propensity score matching

Characteristic	TL (n = 277)	CRT (n = 277)	*p* value
	No. (%)	No. (%)	
Median Age [range] years old	69 [44–92]	69 [39–89]	0.951
Sex			1.000
Male	261 (94.2)	262 (94.6)	
Female	16 (5.8)	15 (5.4)	
Performance status			0.646
0	231 (83.4)	227 (81.9)	
1	40 (14.4)	44 (15.9)	
2	3 (1.1)	5 (1.8)	
3	3 (1.1)	1 (0.4)	
cN			0.420
N0	187 (67.5)	198 (71.5)	
N1	34 (12.3)	25 (9.0)	
N2 [a/b/c]	56 (20.2)	54 (19.5)	
Subsite			0.048
NOS	6 (2.2)	5 (1.8)	
Supraglottic	127 (45.8)	120 (43.3)	
Glottic	131 (47.3)	149 (53.8)	
Subglottic	13 (4.7)	3 (1.1)	

*RT*, radiotherapy; *CRT*, chemoradiotherapy; *TL*, total laryngectomy; *CRT*, chemoradiotherapy; *ICT*, induction chemotherapy, *NOS*, not otherwise specified

Since there were significant differences in the subsites among two groups, next we performed a 1:1 PSMA separately for glottic and supraglottic cancers. One hundred and forty pairs were selected for glottic cancer, and 125 pairs were selected for supraglottic cancer (supplementary Table 1, 2). There was a significant difference in 5-year LCR (95.7% vs. 81.3%, *p* < 0.001) between the TL and CRT groups for glottic cancer, but not in 5-year OS (76.1% vs. 76.8%, *p* = 0.825), 5-year DSS rate (87.8% vs. 83.3%, *p* = 0.307) and 5-year LRFS rate (76.6% vs. 68.4%, *p* = 0.0873) were not significantly different (supplementary Fig. 2). Similarly, in supraglottic cancer, there were significant differences in 5-year LCR (95.7% vs. 83.5%, *p* = 0.00575) and 5-year LRFS (71.6% vs. 55.6%, *p* = 0.0239), but not in 5-year OS (70.9% vs. 66.4%, *p* = 0.826) and 5-year DSS rate (83.6% % vs. 81.3%, *p* = 0.57) (supplementary Fig. 3).

### Patients with T4aN0-2M0 laryngeal cancer

There were 430 patients with T4aN0-2M0 LC (396 men and 34 women; median age, 68 years [range: 38–97 years]). Among them, there were 333, 48, and 49 patients in the TL, CRT, and ICT groups, respectively. Table 3 shows the clinical characteristics of the three groups. The *p* values for age, sex, PS, clinical N classification and subsite were 0.002, 0.803, 0.968, and 0.009, and 0.001, respectively. Older patients tended to be treated with TL, whereas patients with multiple lymph node metastases tended to be treated with ICT. Patients with supraglottic cancer tended to be treated with CRT or ICT.

**Table 3:** Characteristics of patients with T4aN0-2M0 LC treated with TL, CRT, and ICT before 1:1 propensity score matching

Characteristic	TL (n = 333)	CRT (n = 48)	ICT (n = 49)	*p* value
	No. (%)	No. (%)	No. (%)	
Median age [range] Years old	70 [38–97]	65 [46–89]	65.5 [47–81]	0.002
Sex				0.803
Male	308 (92.5)	44 (91.7)	44 (89.8)	
Female	25 (7.5)	4 (8.3)	5 (10.2)	
Performance status				0.968
0	236 (70.9)	36 (75.0)	35 (71.4)	
1	74 (22.2)	11 (22.9)	11 (22.4)	
2	19 (5.7)	1 (2.1)	3 (6.1)	
3	2 (0.6)	0 (0.0)	0 (0.0)	
4	2 (0.6)	0 (0.0)	0 (0.0)	
cN				0.009
N0	189 (56.8)	20 (41.7)	16 (32.7)	
N1	35 (10.5)	5 (10.4)	6 (12.2)	
N2 [a/b/c]	109 (32.7)	23 (47.9)	27 (55.1)	
Subsite				0.001
NOS	24 (7.2)	0 (0.0)	4 (8.2)	
Supraglottic	108 (32.4)	28 (58.3)	25 (51.0)	
Glottic	168 (50.5)	15 (31.2)	19 (38.8)	
Subglottic	33 (9.9)	5 (10.4)	1 (2.0)	

*RT*, radiotherapy; *CRT*, chemoradiotherapy; *TL*, total laryngectomy; *CRT*, chemoradiotherapy; *ICT*, induction chemotherapy, *NOS*, not otherwise specified

**Fig. 5:**
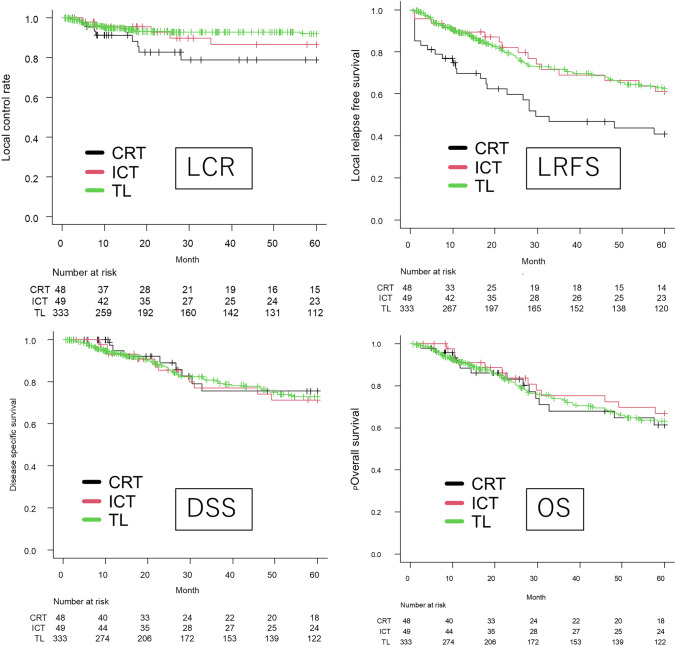
Kaplan–Meier curves of patients with T4aN0-2M0 LC treated with TL, CRT, or ICT before 1:1 propensity score matching. CRT, chemoradiotherapy; ICT, induction chemotherapy; TL, total laryngectomy; LCR, local control rate; LRFS, local recurrence-free survival; DSS, disease-specific survival; OS, overall survival. Black line, CRT group; red line, ICT group; light blue line, TL group

**Fig. 6:**
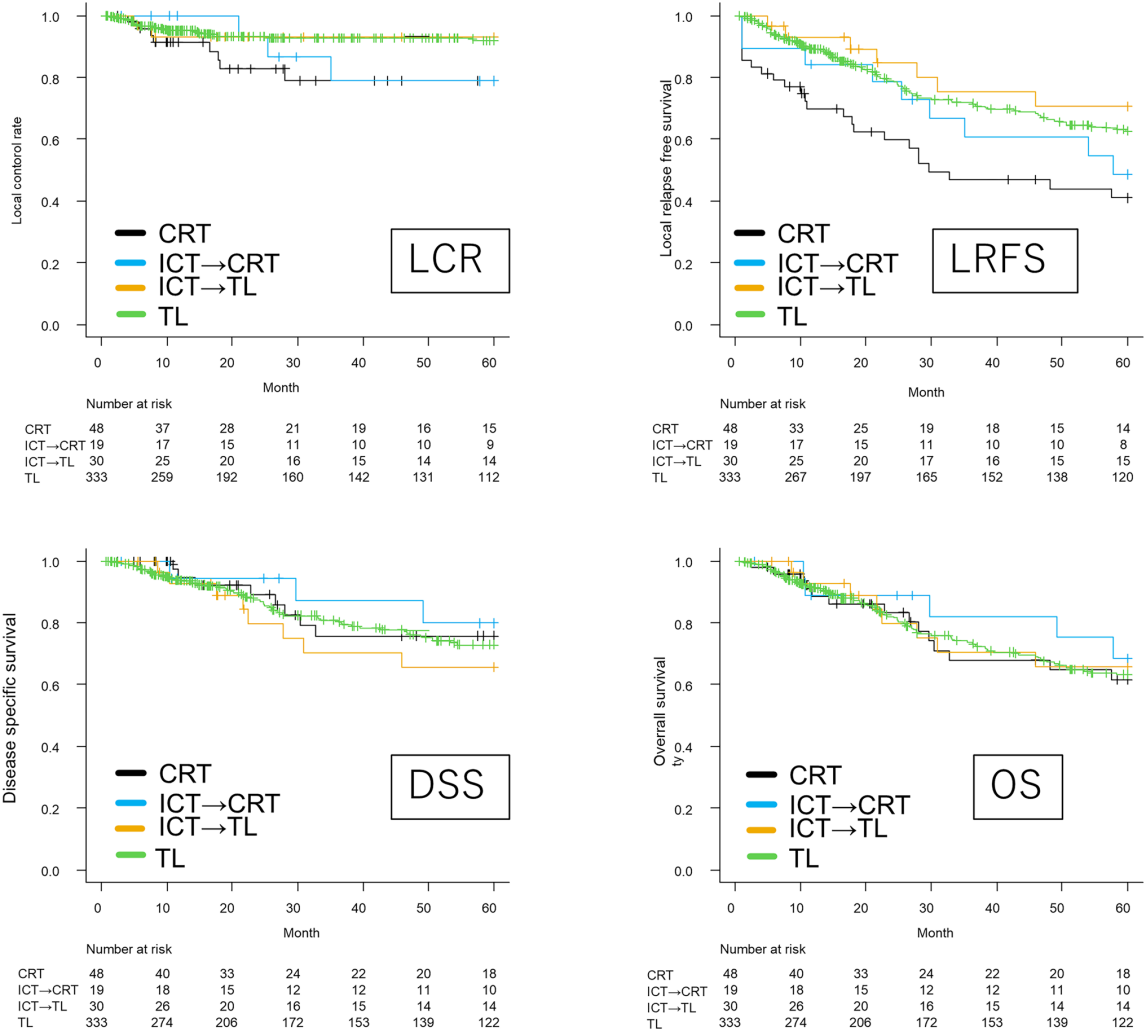
Kaplan–Meier curves of patients with T4aN0-2M0 LC treated with TL, CRT, ICT-TL, or ICT-CRT before 1:1 propensity score matching. CRT, chemoradiotherapy; ICT, induction chemotherapy; TL, total laryngectomy; LCR, local control rate;　LRFS, local recurrence free survival;　DSS, disease specific survival; OS, overall survival. Black line, CRT group; red line, ICT-CRT group; light blue line, ICT-TL group; blue line, TL group

Figures 5 and 6 show the oncological outcomes of patients with T4aN0-2M0 LC. As shown in Fig. 5, there were no significant differences among the TL, CRT and ICT groups in the 5-year OS rate (63.3%, 61.5%, 66.9% *p* = 0.626) or DSS rates (73.0%, 75.7%, 71.4% *p* = 0.966). Compared with the CRT group, the TL group had a better LCR (92.0% vs. 78.9%, *p* = 0.03) and LRFS (62.6% vs. 41.0%, *p* < 0.0001). However, there was no significant difference between the TL and ICT groups in LCR and LRFS (LCR, 92.0% vs. 86.5%, *p* = 0.529; LRFS 62.6% vs. 61.1%, *p* = 0.66).

As shown in Fig. 6, there were no significant differences between the TL and ICT-TL groups in the 5-year OS rate (63.3% vs. 65.7%, *p* = 0.558), DSS rate (73.0% vs. 65.7%, *p* = 0.535), LCR rate (92.0% vs. 93.1%, *p* = 0.881), and LRFS (62.6% vs. 70.6%, *p* = 0.281). Similarly, there were no significant differences between the CRT and ICT-CRT groups in the 5-year OS rate (61.5% vs. 68.4%, *p* = 0.477), DSS rate (75.7% vs. 79.9%, *p* = 0.584), and LCR rate (78.9% vs. 78.8%, *p* = 0.713). However, LRFS of the ICT-CRT tended to be better than that of CRT group (48.7% vs. 41.0%, *p* = 0.225).

Since there was a tendency for multiple lymph node metastases to occur in the ICT group, we compared OS and DSS among the TL, CRT, and ICT groups in T4aN2M0 cases. There were no significant differences in 5-year OS rate (49.5%, 44.5%, 58.4%; CRT vs ICT *p* = 0.357, TL vs ICT *p* = 0.25) and 5-year DSS rate (56.2%, 65.6%, 66.1%, CRT vs ICT *p* = 0.716, TL vs ICT *p* = 0.279). Although no statistically significant difference was observed, the 5-year OS rate tended to be better in the ICT group (supplementary Fig. 1).

**Fig. 7:**
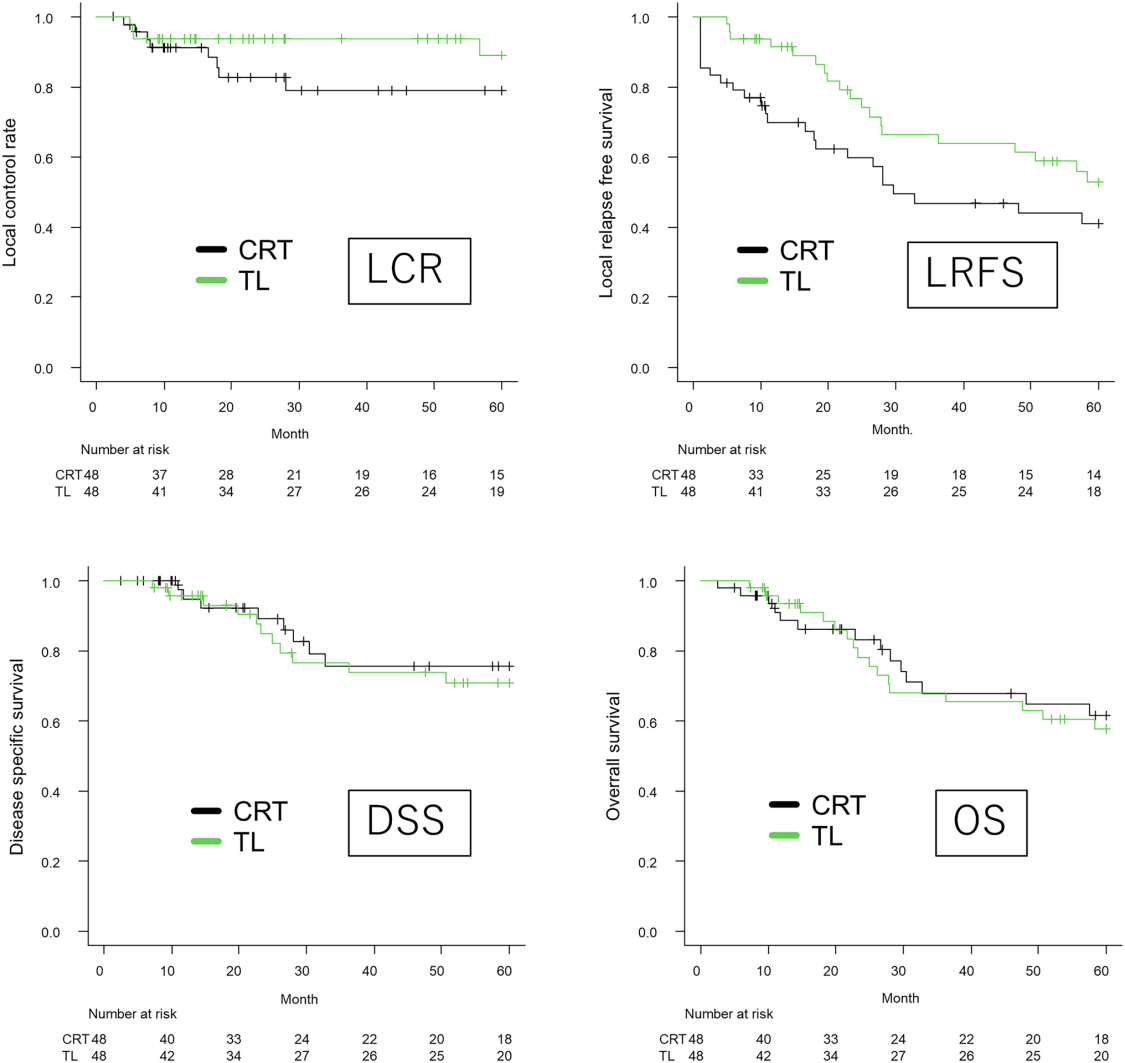
Kaplan–Meier curves of patients with T4aN0-2M0 LC treated with TL or CRT after 1:1 propensity score matching. CRT, chemoradiotherapy; ICT, induction chemotherapy; TL, total laryngectomy; LCR, local control rate;　LRFS, local recurrence-free survival; DSS, disease-specific survival; OS, overall survival. Black line, CRT group; red line, TL group

After excluding the ICT group, 48 pairs were selected using a 1:1 PSMA (Table 4). Figure 7 shows the oncological outcomes of matched patients with T4aN0-2M0 LC in each group. There were significant differences between TL and CRT groups in the 5-year LRFS rate (52.7% vs. 41.0%, *p* = 0.0277). While statistical analysis did not reach significance, 5-year LCR of TL group was better than that of CRT group (89.1 vs. 78.9%, *p* = 0.188). However, there were no significant between-group differences in the 5-year OS (57.7% vs. 61.5%, *p* = 0.764), or 5-year DSS (70.9% vs. 75.7%, *p* = 0.62).

**Table 4:** Characteristics of patients with T4aN0-2M0 LC treated with TL and CRT after 1:1 propensity score matching

Characteristic	TL (n = 48)	CRT (n = 48)	*p* value
	No. (%)	No. (%)	
Median age [range], y	64 [42–85]	65 [46–84]	0.270
Sex			0.677
Male	46 (95.8)	44 (91.7)	
Female	2 (4.2)	4 (8.3)	
Performance Status			0.457
0	40 (83.3)	36 (75.0)	
1	6 (12.5)	11 (22.9)	
2	2 (4.2)	1 (2.1)	
cN			0.558
N0	25 (52.1)	20 (41.7)	
N1	3 (6.2)	5 (10.4)	
N2 [a/b/c]	20 (41.7)	23 (47.9)	
Subsite			0.019
NOS	4 (8.3)	0 (0.0)	
Supraglottic	16 (33.3)	28 (58.3)	
Glottic	24 (50.0)	15 (31.2)	
Subglottic	4 (8.3)	5 (10.4)	

*RT*, radiotherapy; *CRT*, chemoradiotherapy; *TL*, total laryngectomy; *CRT*, chemoradiotherapy; *ICT*, induction chemotherapy, *NOS*, not otherwise specified

Since there were significant differences in the subsites among two groups, a 1:1 PSMA was performed separately for glottic and supraglottic cancers. Fourteen pairs were selected for glottic cancer and 28 pairs for supraglottic cancer (supplementary Table 3, 4). There were no significant differences in 5-year OS (36.7% vs. 69.8%, *p* = 0.1), 5-year DSS rate (58.9% vs. 81.5%, *p* = 0.285), 5-year LRFS rate (36.7% vs. 41.1%, *p* = 0.948), 5-year LCR (100% vs. 70.7%, *p* = 0.0953) between the TL and CRT groups for glottal cancer (supplementary Fig. 4). There were no significant differences in 5-year OS (56.4% vs. 55.5%, *p* = 0.982), 5-year DSS rate (64.0% vs. 75.4%, *p* = 0.399), 5-year LRFS rate (49.7% vs. 44.0%, *p* = 0.187), and 5-year LCR (88.6% vs. 83.4%, *p* = 0.442) between the TL and CRT groups for supraglottic cancer (supplementary Fig. 5).

## Discussion

In this study, we utilized large-scale nationwide clinical data to compare the oncological outcomes of patients with resectable locally advanced LC who underwent TL or CRT. T4b and N3 are occasionally considered as “unresectable” and might be treated with CRT or ICT. Accordingly, to eliminate selection bias in patients with T4b and N3 cancers, we excluded patients with N3 or T4b cancers and focused on those with clearly resectable T3N0-2M0 and T4aN0-2M0 LC.

### Rationale of induction chemotherapy

In this study, no significant intergroup differences in the OS, and DSS in patients with T3N0-2M0 and T4aN0-2M0 LC. This suggests that ICT does not benefit patients with T3-4aN0-2M0 LC with respect to survival. However, the ICT group tended to exhibit significantly more lymph node metastases. ICT may allow enhanced therapeutic effects in patients with multiple lymph node metastases, yielding comparable oncological outcomes as the CRT and TL groups. In terms of laryngeal preservation, the LCR and LRFS were not significantly different between the TL and ICT groups. In addition, the ICT-CRT group tended to have a better LRFS rate than the CRT group, especially in the patients with T4aN0-2M0 LC. These findings further suggest the role of ICT in laryngeal preservation, especially in patients with T4aN0-2M0 LC.

The National Comprehensive Cancer Network guidelines recommend ICT for patients with T3 LC requiring TL and patients with T4a who refuse TL [14]. Accordingly, ICT is administered given its fast response and rapid regression of severe symptoms. However, there is no clear evidence that ICT improves patient survival. Moreover, ICT involves a high risk of serious toxicity in patients with severe comorbidities, which involves a significant mortality rate [15–20]. Furthermore, 20–30% of patients who undergo ICT do not complete CRT [20–22]. Therefore, it is important to carefully evaluate ICT indications for advanced LC with consideration of various factors, including nodal status, organ function, comorbidities, and patient preference.

### Rationale of chemoradiotherapy

To eliminate the impact of ICT, which may have significantly affected survival and laryngeal preservation, we excluded patients who underwent ICT in the PSMA. Subsequently, we performed PSMA in patients with T3N0-2M0 and T4aN0-2M0 LC who were treated with TL or CRT. In patients with T3N0-2M0 LC, there were significant differences in LCR and LRFS between the TL and CRT groups. In the subsite specific study, there were significant differences in LCR for glottic carcinoma and in LCR and LRFS for supraglottic cancer. In patients with T4aN0-2M0 LC, there was a significant between-group difference in LRFS but not in the OS or DSS. However, no significant differences were observed in OS, DSS, LRFS, or LCR by subsite. Taken together, our findings suggest that CRT can be used to treat locally advanced LC without compromising survival compared to TL in patients with T3-4aN0-2M0, regardless of subsites.

TL and CRT have been standard treatments for locally advanced LC, with several meta-analyses comparing these two modalities. Tang et al. found no difference in OS and DFS in patients with advance-stage T3 LC between the TL and CRT groups; however, the TL group had a significantly better OS than the CRT group in patients with T4 LC [7]. A meta-analysis comparing surgical and nonsurgical treatment group found no significant difference in OS in patients with T3 LC [12]. However, in patients with T4 LC, the surgical treatment group had a significantly better OS than the nonsurgical treatment group. A Dutch study based on Netherlands Cancer registry (NCR) data found no significant difference in 5-year OS between the TL and CRT groups in patients with T3 LC. However, the TL group had a better 5-year OS than the CRT group in patients with T4 LC [23]. Additionally, Grover et al. reported that the TL group had significantly better median survival than the CRT group in patients in the T4 LC-based National Cancer Database (NCDB) [11]. Contrastingly, Lee et al. and Shelan et al. reported no difference in OS between the TL and CRT groups in patients with T3 and T4 LC, which is consistent with our findings [24, 25]. This inconsistency may be attributed to the selection criteria for TL and CRT. In NCR and NCDB, only 50.4% and 36% of patients with T4 LC were treated with TL, respectively [11, 23]. Contrastingly, the studies conducted by Lee et al. and Shelan et al., as well as the present study, 77–84% of patients with T4 LC underwent TL. These results suggest that careful patient selection for CRT, with consideration for organ function sufficient to tolerate chemotherapy as well as tumor volume, can yield oncological outcomes comparable to those of TL.

Another possible reason for the inconsistent findings may be salvage surgery. Since up to 50% of patients with advanced SCC of the head and neck recur after CRT, salvage surgery with curative intent plays a crucial role in these cases [26–33]. Although patients with unresectable local recurrence have a poor prognosis, salvage surgery for resectable recurrences can improve prognosis [31]. However, the stage progression at initial treatment is negatively correlated with the survival rate after salvage surgery for recurrence. In Japan, the rate of salvage surgery for local–regional recurrence after CRT for LC is 70–88% [27, 34], while it is much lower in other countries (61–71%) [24, 30, 33]. In our study, the CRT group had a significantly lower LRFS than the TL group. However, the CRT group had a comparable OS and DSS as the TL group. Taken together, these findings suggest that salvage surgery at the time of local recurrence in the CRT group may have contributed to the improved survival.

### Limitations

This study has several limitations. First, we did not include detailed information regarding salvage treatments for local recurrence. Therefore, we did not perform accurate assessment of laryngeal preservation. Second, the data did not include detailed information on the regimens used for CRT and ICT. This may have led to differences in treatment efficacy. Third, the results of propensity score matching, especially for patients with T4aN0-2M0 LC (n = 48), might not reflect accurate prognosis due to the reduced sample size. Finally, since we utilized a national database, we could not obtain detailed information, including tumor volume and the presence of other cancers or comorbidities, which may be relevant to treatment selection. Nonetheless, it would be difficult to include such detailed information in meta-analyses. A large-scale multicenter study with more detailed information is warranted to provide more definitive conclusions.

## Conclusion

Our findings indicated that ICT showed no apparent benefits in patients with T3N0-2M0 and T4aN0-2M0 LC. In PSMA, there no significant between-group differences in OS and DSS across all patients. These results suggest that CRT may not compromise survival in patients with T3-4aN0-2M0 LC.

## Supplementary Information

Below is the link to the electronic supplementary material.

Supplementary file1 (TIF 646 KB)

Supplementary file2 (TIF 591 KB)

Supplementary file3 (TIF 590 KB)

Supplementary file4 (TIF 561 KB)

Supplementary file5 (TIF 570 KB)

Supplementary file6 (DOCX 15 KB)

Supplementary file7 (DOCX 16 KB)

Supplementary file8 (DOCX 15 KB)

Supplementary file9 (DOCX 16 KB)
