# Supplementary figures and images for: Survival outcomes of the patients with advanced laryngeal squamous cell carcinoma treated with chemoradiotherapy and total laryngectomy based on reports of head and neck cancer registry of Japan
Source: Int J Clin Oncol. 2026 May 7;31(7):1201–14. doi: 10.1007/s10147-025-02938-4 (PMC13303432; doi:10.1007/s10147-025-02938-4)

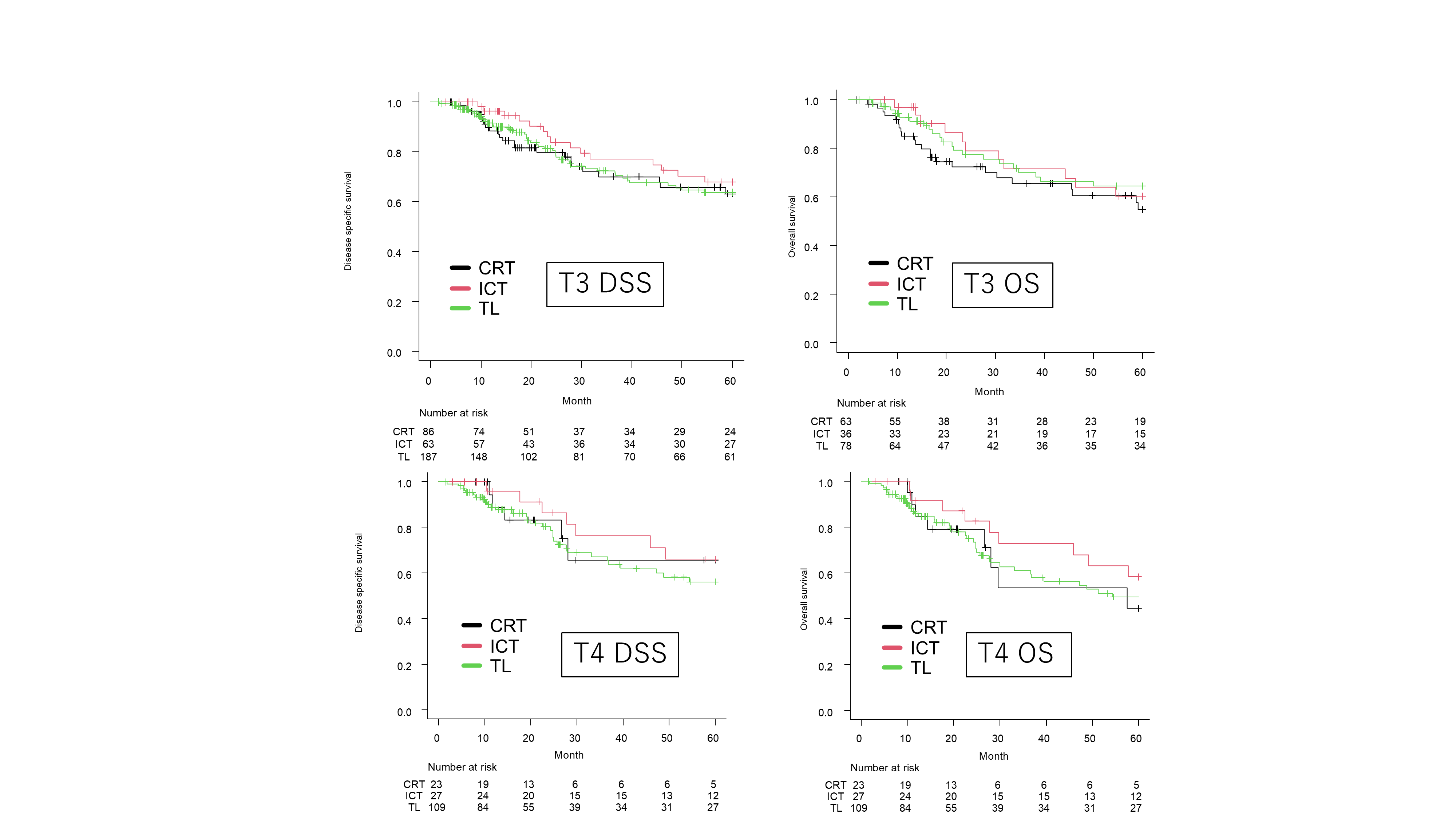

Supplement: Supplementary file 1 — Supplementary file1 (TIF 646 KB) [file 10147_2025_2938_MOESM1_ESM.tif]

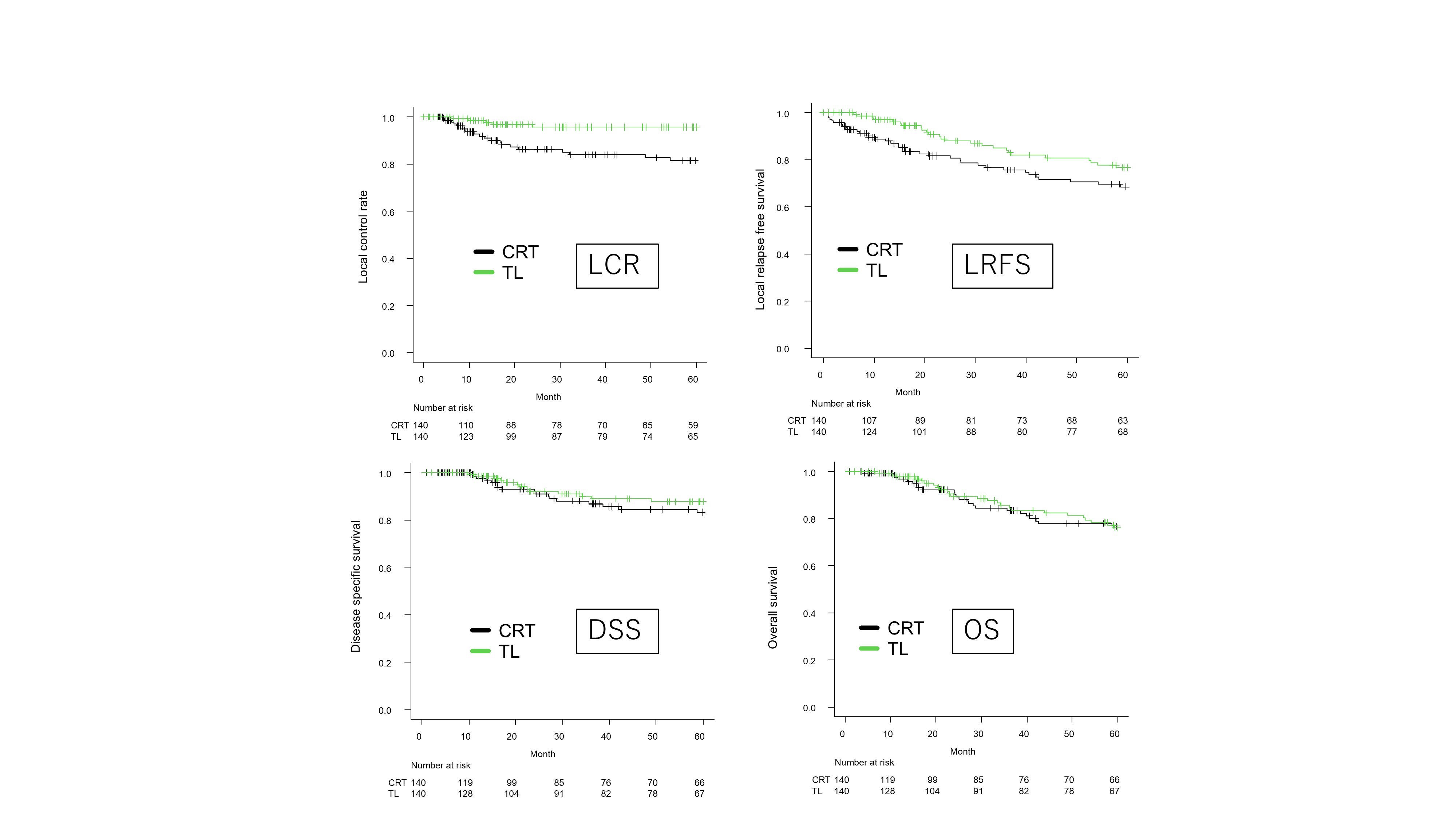

Supplement: Supplementary file 2 — Supplementary file2 (TIF 591 KB) [file 10147_2025_2938_MOESM2_ESM.tif]

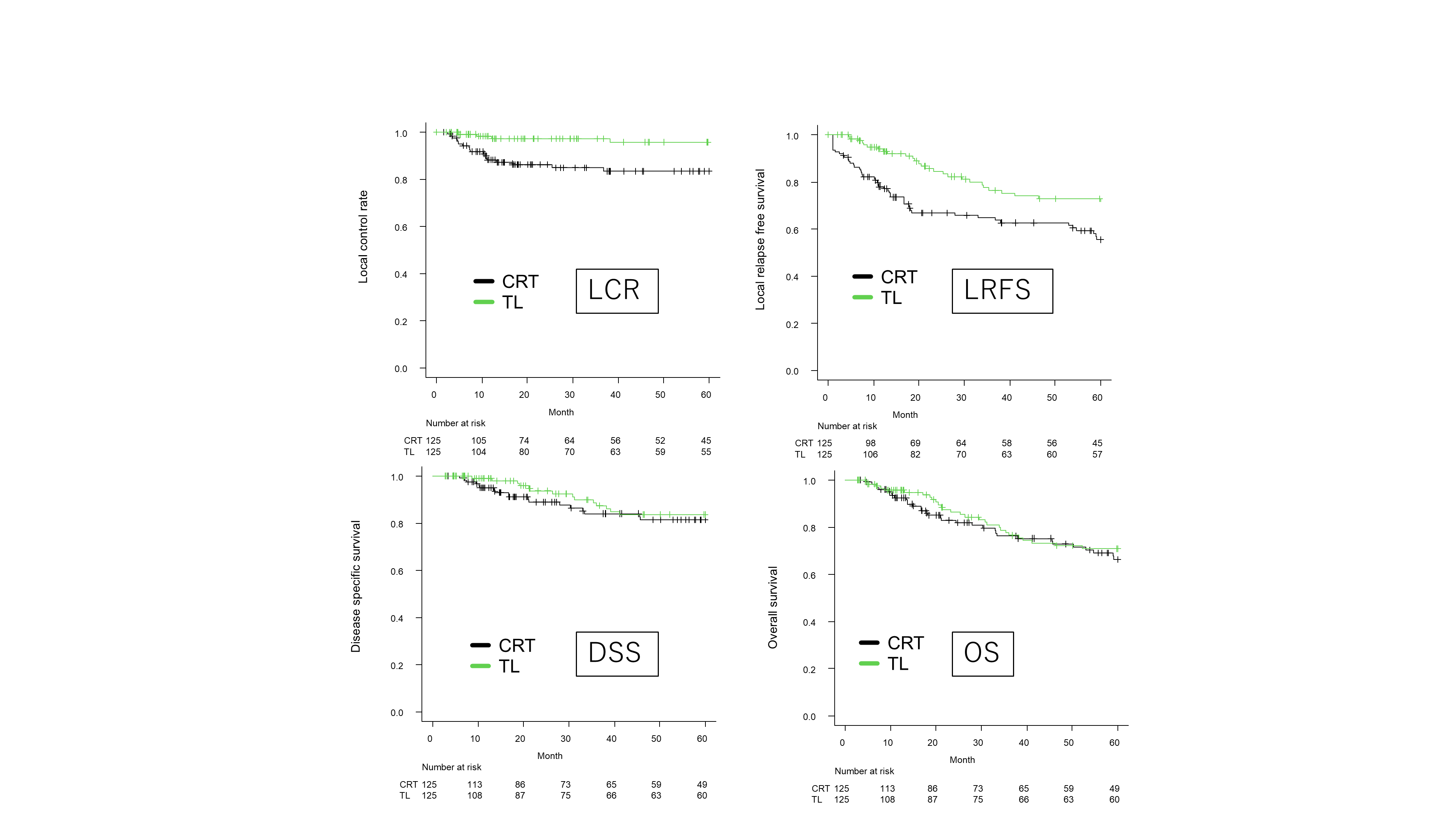

Supplement: Supplementary file 3 — Supplementary file3 (TIF 590 KB) [file 10147_2025_2938_MOESM3_ESM.tif]

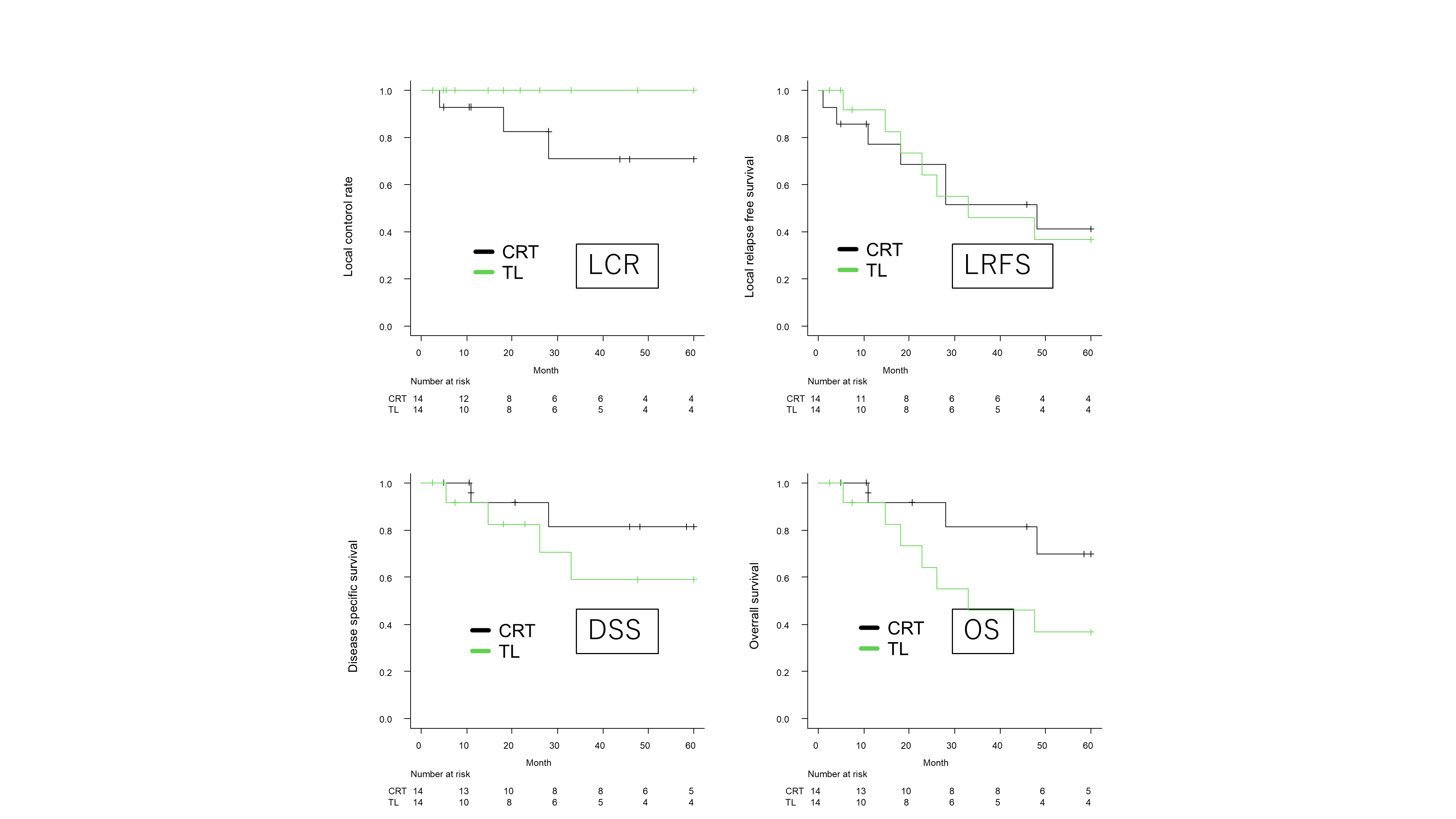

Supplement: Supplementary file 4 — Supplementary file4 (TIF 561 KB) [file 10147_2025_2938_MOESM4_ESM.tif]

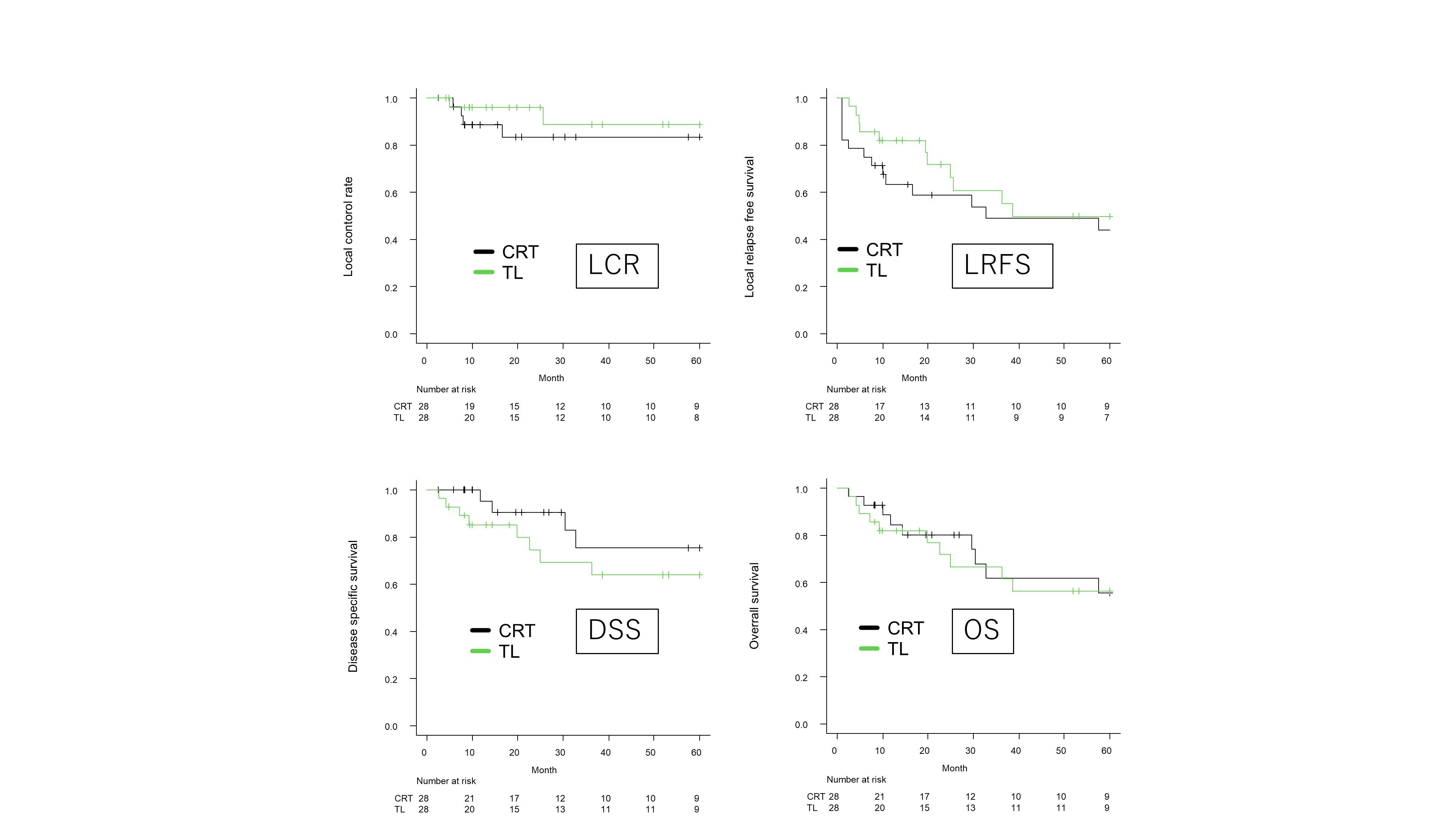

Supplement: Supplementary file 5 — Supplementary file5 (TIF 570 KB) [file 10147_2025_2938_MOESM5_ESM.tif]
