# Supplementary material for: Survival outcomes of the patients with advanced laryngeal squamous cell carcinoma treated with chemoradiotherapy and total laryngectomy based on reports of head and neck cancer registry of Japan
Source: Int J Clin Oncol. 2026 May 7;31(7):1201–14. doi: 10.1007/s10147-025-02938-4 (PMC13303432; doi:10.1007/s10147-025-02938-4)
Supplement: Supplementary file 6 — Supplementary file6 (DOCX 15 KB) [file 10147_2025_2938_MOESM6_ESM.docx]

Supplementary Table 1

| Characteristic | TL (n= 140) | CRT (n= 140) | P value |
| --- | --- | --- | --- |
|  | No. (%) | No. (%) |  |
| Median Age [range]  years old | 69 [40-86] | 69 [39-82] | 0.857 |
| Sex |  |  | 0.501 |
| Male | 137 (97.9) | 134 (95.7) |  |
| Female | 3 (2.1) | 6 (4.3) |  |
| Performance status |  |  | 0.741 |
| 0 | 109(77.9) | 112(80.0) |  |
| 1 | 27 (19.3) | 26 (18.6) |  |
| 2 | 4 (2.9) | 2 (1.4) |  |
| cN |  |  | 0.671 |
| N0 | 123 (87.9) | 127(90.7) |  |
| N1 | 10 (7.1) | 6 (4.3) |  |
| N2[a/b/c] | 7 (5.0) | 7 (5.0) |  |
