# Supplementary material for: Survival outcomes of the patients with advanced laryngeal squamous cell carcinoma treated with chemoradiotherapy and total laryngectomy based on reports of head and neck cancer registry of Japan
Source: Int J Clin Oncol. 2026 May 7;31(7):1201–14. doi: 10.1007/s10147-025-02938-4 (PMC13303432; doi:10.1007/s10147-025-02938-4)
Supplement: Supplementary file 7 — Supplementary file7 (DOCX 16 KB) [file 10147_2025_2938_MOESM7_ESM.docx]

Supplementary Table 2

| Characteristic | TL (n= 125) | CRT (n= 125) | P value |
| --- | --- | --- | --- |
|  | No. (%) | No. (%) |  |
| Median Age [range]  years old | 69 [44-91] | 69 [45-89] | 0.866 |
| Sex |  |  | 1.000 |
| Male | 115 (92.0) | 116 (92.8) |  |
| Female | 10 (8.0) | 9 (7.2) |  |
| Performance status |  |  | 0.955 |
| 0 | 102(81.6) | 105 (84.0) |  |
| 1 | 19 (15.2) | 16 (12.8) |  |
| 2 | 3 (2.4) | 3 (2.4) |  |
| 3 | 1 (0.8) | 1 (0.8) |  |
| cN |  |  | 0.416 |
| N0 | 55 (44.0) | 59 (47.2) |  |
| N1 | 25 (20.0) | 17 (13.6) |  |
| N2[a/b/c] | 45 (36.0) | 49 (39.2) |  |
