# Supplementary material for: Survival outcomes of the patients with advanced laryngeal squamous cell carcinoma treated with chemoradiotherapy and total laryngectomy based on reports of head and neck cancer registry of Japan
Source: Int J Clin Oncol. 2026 May 7;31(7):1201–14. doi: 10.1007/s10147-025-02938-4 (PMC13303432; doi:10.1007/s10147-025-02938-4)
Supplement: Supplementary file 8 — Supplementary file8 (DOCX 15 KB) [file 10147_2025_2938_MOESM8_ESM.docx]

Supplementary Table 3

| Characteristic | TL (n= 14) | CRT (n= 14) | P value |
| --- | --- | --- | --- |
|  | No. (%) | No. (%) |  |
| Median Age [range]  years old | 63.5 [56-84] | 63 [52-89] | 0.840 |
| Sex |  |  | 1.000 |
| Male | 12 (85.7) | 13 (92.9) |  |
| Female | 2 (14.3) | 1 (7.1) |  |
| Performance status |  |  | 0.098 |
| 0 | 13(92.9) | 10 (71.4) |  |
| 1 | 0 (0.0) | 4 (28.6) |  |
| 2 | 1 (7.1) | 0 (0.0) |  |
| cN |  |  | 1.000 |
| N0 | 10 (71.4) | 9 (64.3) |  |
| N1 | 0 (0.0) | 1 (7.1) |  |
| N2[a/b/c] | 4 (28.6) | 4 (28.6) |  |
