# Supplementary material for: Survival outcomes of the patients with advanced laryngeal squamous cell carcinoma treated with chemoradiotherapy and total laryngectomy based on reports of head and neck cancer registry of Japan
Source: Int J Clin Oncol. 2026 May 7;31(7):1201–14. doi: 10.1007/s10147-025-02938-4 (PMC13303432; doi:10.1007/s10147-025-02938-4)
Supplement: Supplementary file 9 — Supplementary file9 (DOCX 16 KB) [file 10147_2025_2938_MOESM9_ESM.docx]

Supplementary Table 4

| Characteristic | TL (n= 28) | CRT (n= 28) | P value |
| --- | --- | --- | --- |
|  | No. (%) | No. (%) |  |
| Median Age [range]  years old | 70.0 [49-87] | 65.5 [49-87] | 0.379 |
| Sex |  |  | 1.000 |
| Male | 26 (92.9) | 25 (89.3) |  |
| Female | 2 (7.1) | 3 (10.7) |  |
| Performance status |  |  | 0.392 |
| 0 | 24(85.7) | 20 (71.4) |  |
| 1 | 3 (10.7) | 7 (25.0) |  |
| 2 | 1 (3.6) | 1 (3.6) |  |
| cN |  |  | 1.000 |
| N0 | 8 (28.6) | 8 (28.6) |  |
| N1 | 4 (14.3) | 4 (14.3) |  |
| N2[a/b/c] | 16 (57.1) | 16 (57.1) |  |
